# Parental perceptions and the 5C psychological antecedents of COVID-19 vaccination during the first month of omicron variant surge: A large-scale cross-sectional survey in Saudi Arabia

**DOI:** 10.3389/fped.2022.944165

**Published:** 2022-08-16

**Authors:** Shuliweeh Alenezi, Mohammed Alarabi, Ayman Al-Eyadhy, Fadi Aljamaan, Iffat Elbarazi, Basema Saddik, Khalid Alhasan, Rasha Assiri, Rolan Bassrawi, Fatimah Alshahrani, Nasser S. Alharbi, Amel Fayed, Sheikh Minhaj Ahmed, Rabih Halwani, Khaled Saad, Sarah Alsubaie, Mazin Barry, COVID-19 Saudi Research Consortium, Ziad A. Memish, Jaffar A. Al-Tawfiq, Mohamad-Hani Temsah

**Affiliations:** ^1^College of Medicine, King Saud University, Riyadh, Saudi Arabia; ^2^Department of Psychiatry, College of Medicine, King Saud University Medical City, King Saud University, Riyadh, Saudi Arabia; ^3^Pediatric Kidney Transplant, Organ Transplant Center of Excellence, King Faisal Specialist Hospital and Research Center, Riyadh, Riyadh, Saudi Arabia; ^4^Critical Care Department, King Saud University Medical City, King Saud University, Riyadh, Saudi Arabia; ^5^Institute of Public Health, College of Medicine and Health Sciences, United Arab Emirates University, AlAin, United Arab Emirates; ^6^Department of Family and Community Medicine, College of Medicine, University of Sharjah, Sharjah, United Arab Emirates; ^7^Sharjah Institute of Medical Research, University of Sharjah, Sharjah, United Arab Emirates; ^8^Department of Basic Medical Sciences, College of Medicine, Princess Nourah Bint Abdulrahman University, Riyadh, Saudi Arabia; ^9^Division of Infectious Diseases, Department of Internal Medicine, King Saud University Medical City, King Saud University, Riyadh, Saudi Arabia; ^10^Clinical Sciences Department, College of Medicine, Princess Nourah Bint Abdulrahman University, Riyadh, Saudi Arabia; ^11^Division of Pediatric Critical Care, Department of Pediatrics, Lilavati Hospital and Research Center, Mumbai, India; ^12^Department of Clinical Sciences, College of Medicine, University of Sharjah, Sharjah, United Arab Emirates; ^13^Pediatric Department, Faculty of Medicine, Assiut University, Assiut, Egypt; ^14^Division of Infectious Diseases, Faculty of Medicine, University of Ottawa, Ottawa, ON, Canada; ^15^King Saud Medical City, Ministry of Health and Alfaisal University, Riyadh, Saudi Arabia; ^16^Hubert Department of Global Health, Emory University, Atlanta, GA, United States; ^17^Specialty Internal Medicine and Quality Department, Johns Hopkins Aramco Healthcare, Dhahran, Saudi Arabia; ^18^Infectious Disease Division, Department of Medicine, Indiana University School of Medicine, Indianapolis, IN, United States; ^19^Infectious Disease Division, Department of Medicine, Johns Hopkins University School of Medicine, Baltimore, MD, United States

**Keywords:** COVID-19, SARS-CoV-2 among general population, omicron variant worries, vaccination hesitancy, 5C Scale

## Abstract

**Background:**

With the rapid surge of SARS-CoV-2 Omicron variant, we aimed to assess parents' perceptions of the COVID-19 vaccines and the psychological antecedents of vaccinations during the first month of the Omicron spread.

**Methods:**

A cross-sectional online survey in Saudi Arabia was conducted (December 20, 2021-January 7, 2022). Convenience sampling was used to invite participants through several social media platforms, including WhatsApp, Twitter, and email lists. We utilized the validated 5C Scale, which evaluates five psychological factors influencing vaccination intention and behavior: confidence, complacency, constraints, calculation, and collective responsibility.

**Results:**

Of the 1,340 respondents, 61.3% received two doses of the COVID-19 vaccine, while 35% received an additional booster dose. Fify four percentage were unwilling to vaccinate their children aged 5–11, and 57.2% were unwilling to give the additional booster vaccine to children aged 12–18. Respondents had higher scores on the construct of collective responsibility, followed by calculation, confidence, complacency, and finally constraints. Confidence in vaccines was associated with willingness to vaccinate children and positively correlated with collective responsibility (*p* < 0.010). Complacency about COVID-19 was associated with unwillingness to vaccinate older children (12–18 years) and with increased constraints and calculation scores (*p* < 0.010). While increasing constraints scores did not correlate with decreased willingness to vaccinate children (*p* = 0.140), they did correlate negatively with confidence and collective responsibility (*p* < 0.010).

**Conclusions:**

The findings demonstrate the relationship between the five antecedents of vaccination, the importance of confidence in vaccines, and a sense of collective responsibility in parents' intention to vaccinate their children. Campaigns addressing constraints and collective responsibility could help influence the public's vaccination behavior.

## Introduction

The health sequelae of the COVID-19 pandemic are enormous. Since early 2020, SARS-CoV-2 has infected millions of individuals worldwide, killing 4.5 million people and causing ongoing health challenges (1, 2). The increasing burden of survivorship is significant as well, in terms of the complexity of long-term health effects and the number of people affected with post-COVID-19 sequalae (3–5). The scientific efforts to develop and provide vaccines against COVID-19 in mid to late 2020 provided the opportunity to modify the course of the pandemic (6). Vaccines offered protection against the raging virus, lessened the burden of illness if the virus was contracted, and reduced COVID-19-related mortality (7–9). Vaccination of 60–70% of the population was assumed to provide the necessary herd immunity against the virus (10).

Nevertheless, when vaccines became available, the public were overwhelmed with information about the different vaccines. While available vaccines ranged from traditional vaccine types such as adenovirus vectors to emerging technologies such as messenger RNA (mRNA) vaccines (11–13), all vaccines demonstrated their efficacy and safety (14). Not surprisingly, people differed in their reaction to this unprecedented situation. Some were willing to be vaccinated while others were more hesitant and still questioning the vaccines safety and the trustworthiness of information regarding their efficacy (15–17). In the Kingdom of Saudi Arabia (KSA), parents were generally positive toward children's COVID-19 vaccination, but compared to routine vaccination, they were more hesitant toward COVID-19 vaccination (6 vs. 27%) (18). Other studies showed that 42.8% of parents reported COVID-19 vaccine hesitancy, with similar hesitancy rates among parents of children with chronic disorders (19, 20).

Worries about the COVID-19 vaccine and predictors of vaccination acceptance have been studied in different populations since the vaccines were introduced (21–24). These studies showed that, among the public, worries about the trustworthiness of authorities and safety of the vaccines predicted unwillingness to be vaccinated, while certain sociodemographic factors, such as higher education, predicted higher acceptance of the vaccine. Among healthcare workers (HCWs), despite the majority accepting vaccination against COVID-19 (25), a substantial minority were hesitant or unwilling to be vaccinated. The reasons reported included fears about potential side effects and perceptions, that the vaccines' approval was rushed during the initial stages of vaccine roll out (26, 27). People faced another challenging choice when mRNA COVID-19 vaccines were approved for teenagers and children as young as 5 years of age (28, 29). Studies exploring parents' and caregivers' attitudes toward vaccinating their children against COVID-19 reveal that a substantial proportion are hesitant to vaccinate them, especially feeling not enough information is available about the safety and efficacy of those vaccines for this age group (30–33).

Unfortunately, global efforts to contain the pandemic were hindered by the emergence of multiple variants of COVID-19 and their spread during the period from late 2020 to late 2021 (34). Despite concerns over the efficacy of available vaccines against emerging variants (35–37), current evidence suggests that the available COVID-19 vaccines remain effective especially after booster doses (38–41). The most recent variant of concern in the evolving pandemic is the strain that was first reported in November 2021 and subsequently named Omicron (42). This strain is more transmissible than previous variants and is currently spreading across the globe, causing a rise in COVID-19 cases worldwide and worries among HCWs (43–46). With the possibility of a prolonging pandemic owing to the rapid spread of the Omicron variant, it is warranted to explore parents' attitudes and perceptions toward COVID-19 vaccination.

Researchers have developed and utilized various tools to help them explore people's willingness and hesitancy to being vaccinated, including during the COVID-19 pandemic (47–50). The 5C Scale provides an in-depth exploration of the psychological antecedents of vaccinations (51). The scale assesses an individual's trust in vaccines and the systems that provide it (confidence), the lack of perception of diseases as high risk (complacency), structural and psychological barriers to vaccination (constraints), efforts in searching for information (calculation), and willingness to protect others (collective responsibility). Except for confidence and collective responsibility, the other psychological antecedents all predict negative vaccination attitude and unwillingness to be vaccinated. The 5C scale was originally available in English and German but has since been adapted for use in other languages such as Arabic (51, 52). Recently, the scale has been utilized to assess vaccine behavior amidst the currently evolving COVID-19 pandemic (53–55). A large multinational study in the Arab world demonstrated that gender, country of residence, and education amongst other factors were associated with changes in the psychological antecedents of vaccination against COVID-19 as assessed by the 5C scale (56).

The first case of Omicron COVID-19 in the Kingdom Saudi Arabia (KSA) was reported on the first of December 2021 (57). By January 2022, the daily count of COVID-19 cases in KSA reached a new record unprecedented during the pandemic (1). Parents in KSA reported less worry about the Omicron variant compared to the Delta variant (58). The aim of this study is to assess people's perceptions of COVID-19 vaccines and how the 5C psychological antecedents of vaccinations are associated with their willingness to vaccinate their children considering the current spread of the Omicron variant of COVID-19.

## Methods

### Study design and settings

A cross-sectional survey was conducted among adults in KSA between December 20, 2021to January 7, 2022.

### Study population

The inclusion criteria included residing in Saudi Arabia, being an Adult (Age >18 years), and having at least one child. Assuming 50% of the subjects would have the outcome of interest, the sample size desired to detect the true prevalence of the outcome with 95% confidence and a margin of error of 5%, the minimum sample size was 386 subjects.

### Sampling procedure

Convenience sampling was used to invite participants through several social media platforms, including WhatsApp groups Twitter posts, and email lists. The questionnaire was distributed electronically through SurveyMonkey. We reached out to potential study participants once, to avoid the hassle in the era of “survey fatigue,” as the literature reported that the rise in surveys distribution during the COVID-19 pandemic has led to such survey fatigue, with reduced response rates, and lesser data collection quality (59).

### Survey tool and outcome measures

The survey was adapted from the previously published reports on COVID-19 parental and HCWs, with modifications related to the new SARS-CoV-2 Omicron variant (36, 60–63). The final copy of the survey was agreed upon by the research team who reviewed it for language accuracy and for clarity. The survey includes the following sections: Sociodemographic variables, the parents' and their children's COVID-19 and vaccination history, willingness to vaccinate children and causes of possible refusal, sources of information on COVID-19, and an estimate of their family's commitments to universal precautionary measures against COVID-19 using a 1–5 Likert scale with higher scores corresponding to higher commitment. Finally, the scale contained the Arabic 5C scale assessing the psychological antecedents of vaccination.

The 5C is a validated psychometric scale that assesses psychological antecedents of vaccination. It consists of five domains and includes 15 questions distributed among five sub-scales. The first sub-scale measures confidence in the efficacy and safety of the vaccines, and the healthcare system decision-makers, authorities, and healthcare professionals are involved in providing them. The second sub-scale assesses complacency, which refers to the risk perceptions toward vaccine-preventable diseases and whether so vaccination is deemed unnecessary preventive action. The third sub-scale considers barriers related to physical availability, affordability and willingness to pay, geographical accessibility, and health literacy apperceptions of immunization service. The fourth sub-scale assesses calculation which explores individuals' engagement in extensive information searching of vaccination and disease risks. The last sub-scale considers collective responsibility, which aims to provide insights into an individual's willingness to protect others by one's vaccination using herd immunity. The questionnaire was validated in Arabic earlier by a research group from different Arab countries who could determine the cut-off score for each domain to predict COVID-19 vaccine acceptance or hesitancy (51, 63).

### Ethical standards

Participants were informed in the beginning of the survey about the purpose of this study and that their participation was voluntary. Their information did not include any personal identifiers and their responses were completely anonymous. The Institutional Review Board at King Saud University approved the study (21/01139/IRB). Informed consent to participate in the study was incorporated into the first page of the survey (Supplementary material 1).

### Statistical analysis

Descriptive statistics, including means and standard deviations were used to describe continuous variables, and frequencies and percentages were used to summarize categorically measured variables. Multiple response/dichotomies analysis was applied to multiple option questions. Histograms and the Kolmogrov-Smirnov tests of Normality were used to assess the statistical normality assumption of metric variables and the Levene's test assessed the statistical equality of variance assumptions for metric variables.

The descriptive analysis of the respondents' psychological antecedents of vaccination as measured by the 5C Scale was obtained by the means and standard deviations for each of the five subscales which correspond to the five constructs comprising the 5C scale. In order to calculate these subscale scores, the scale items were grouped into their respective constructs according to the 5C Scale manual, and the subscale scores were computed accordingly. Each item of the 5C scale is measured on a 1-7 Likert scale with higher scores corresponding to higher agreement to the item statement, while each computed subscale score ranges from 1 to 7, with higher scores corresponding to a higher level of applicability of the psychological antecedent construct to the respondent. Cronbach's alpha test of reliability was applied to assess the internal consistency of the 5C scale constructs. Additionally, Confirmatory Factor Analysis with the maximum likelihood estimation was applied to the 5C items with Parallel Analysis and the tests of unidimensionality (56).

Multivariate Linear Regression Analysis was applied to each of the five subscales of the 5C scale regressing them against parents' sociodemographic characteristics, reported COVID-19 history, willingness to vaccinate their children, overall Commitment to Universal precautions, and reported sources of information about COVID-19.

The association between these factors and the computed 5C subscale scores was expressed as unstandardized beta coefficients and 95% confidence intervals. Data were analyzed using the Statistical Package for the Social Sciences (SPSS), Version 21.0 (IBM Corp., Armonk, New York, USA). The Stand-Alone FACTOR program [release 10.09.01, (64)] was used for the parallel analysis and the tests of dimensionality of the 5C scale and reproduced the same dimensions as previous studies on the Arabic 5C scale (52). The statistical significance level was considered at 0.050 level.

## Results

Among 1,938 who started the survey, we included the 1,340 who completed the 5C questions from parents residing in Saudi Arabia. Supplementary Table A1 shows the sociodemographic characteristics of the acquired sample. Most participants were female, the dominant age group was 35–55 years, and the vast majority had a university degree.

A quarter of respondents (25.7%) reported being infected with COVID-19 without hospitalization, while (1.5%) reported required hospitalization. (35.4%) reported at least one family member has had COVID-19 infection but did not require hospitalization, (1.8%) required hospital admission, and (0.6%) required intensive medical care admission.

### Psychological antecedents of vaccination

**Figure 1:**
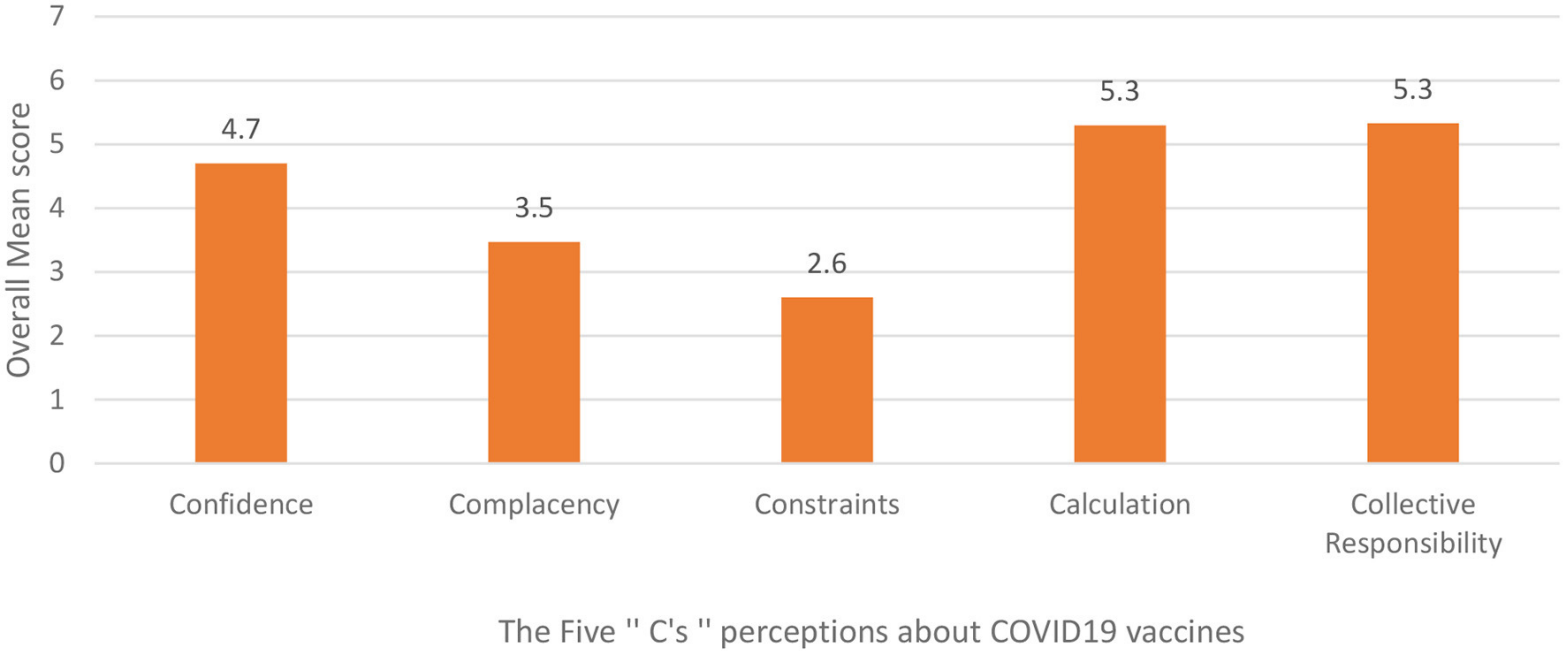
Participants' overall mean perceptions of the 5C scale.

Figure 1 illustrates the mean scores of each construct of the 5C Scale. The internal consistency measure for each of the subscales was good, with a Cronbach's alpha ranging from 0.71 for Constraints to 0.87 for Confidence (see Supplementary Tables A2, A3).

### Correlation between the 5C psychological antecedents of vaccination

Supplementary Table A4 displays the correlation analysis between the computed scores for each of the constructs comprising the 5C scale. The parents' score for confidence correlated significantly and negatively with their score on the Complacency construct. Confidence was similarly negatively correlated with perceived Constraints to vaccination but was less substantially correlated with Calculation, illustrating the negative relationship between these antecedents of vaccination and confidence in vaccines.

Collective responsibility score was positively and strongly correlated with confidence in vaccines, and negatively and substantially correlated with the constructs of complacency and constraints. Finally, we calculated the overall commitment to universal precautionary measures against COVID-19, the overall score was positively correlated with confidence in vaccines and perceived collective responsibility, and negatively correlated with complacency, constraints, and calculation.

### Factors associated with the 5C psychological antecedents of vaccination

#### Confidence

Table 1 displays the multivariate linear regression analysis of parents' confidence in vaccines. Parents' confidence in vaccines is higher in parents belonging to older age groups (>55 years of age). Having a child between the ages of 5–11 years was, on the other hand, associated with decreased confidence in vaccines by the parent. Also associated with decreased confidence were higher complacency and constraints construct scores. Calculation did not seem to be correlated significantly with confidence whereas the association with collective responsibility was significant. This indicated that higher collective responsibility was associated with increased confidence in vaccines. Not surprisingly, the parents' willingness to vaccinate their children in both age groups (5–11 and 12–18 years) was associated with higher confidence, and use of the MOH website as a source of information on vaccines. On the other hand, reporting the use of videos such as YouTube as a source of information was associated with a significant decrease in confidence in vaccines. Finally, parents who reported having been previously infected with COVID-19 had lower confidence in vaccines.

**Table 1:** Multivariate linear regression analysis of parents' confidence as scored by the 5C scale.

	**Unstandardized beta coefficients**	**95.0% CI for B**		***p*-value**
		**Lower bound**	**Upper bound**	
(Constant)	2.102	1.636	2.567	<0.001
Gender = Male	0.114	−0.001	0.229	0.052
Age ≧55 years	0.205	0.017	0.394	0.033
Has children aged 5–11 years	−0.147	−0.278	−0.015	0.028
Has children with chronic physical/mental illness	−0.175	−0.355	0.004	0.055
*Mean complacency score*	−0.059	−0.108	−0.010	0.018
*Mean constraints score*	−0.097	−0.151	−0.042	0.001
*Mean calculation score*	−0.019	−0.063	0.025	0.391
*Mean collective responsibility score*	0.528	0.477	0.580	<0.001
Willingness to vaccinate child (aged 5–11 years)	0.590	0.447	0.734	<0.001
Willingness to vaccinate child (aged 12–18 years)	0.308	0.168	0.447	<0.001
Parent previously infected with COVID-19	−0.220	−0.348	−0.096	0.001
Use of MOH website as source of information	0.220	0.074	0.366	0.003
Use of WHO website as source of information	−0.106	−0.223	0.011	0.076
Use of videos such as YouTube as source of information	−0.202	−0.386	−0.019	0.030
Use of medical articles as a source of information	−0.117	−0.237	0.003	0.056

*Dependent Variables (DV) = mean Confidence Subscale Score. Model R-squared = 0.604, adjusted R-squared = 0.60*.

#### Complacency

Multivariate linear regression analysis of the participants' complacency of the COVID-19 is displayed in Table 2. The model showed that having a family member with previous COVID-19 infection or not taking the annual flu vaccine were associated with higher complacency. Confidence in vaccines and reported collective responsibility were both associated significantly with lower complacency, whereas higher constraints and calculation constructs scores were associated with higher complacency. Another factor for lower complacency was the parent's willingness to vaccinate their child aged 12–18 years but not younger children (5–11 years).

**Table 2:** Multivariate linear regression analysis of parents' complacency as scored by the 5C Scale.

	**Unstandardized beta coefficients**	**95.0% Cl for beta**		***p*-value**
		**Lower bound**	**Upper bound**	
(Constant)	3.081	2.506	3.657	<0.001
Gender = Male	−0.049	−0.178	0.079	0.452
Age group	0.028	−0.043	0.098	0.443
Parent's educational level: Higher postgraduate studies	0.182	−0.012	0.375	0.065
Family member previously infected with COVID-19	0.133	0.029	0.237	0.013
Takes annual seasonal flu vaccine = No	0.169	0.036	0.302	0.013
*Mean confidence score*	−0.067	−0.126	−0.009	0.024
*Mean constraints score*	0.348	0.291	0.404	<0.001
*Mean calculation score*	0.158	0.112	0.205	<0.001
Mean collective responsibility score	−0.248	−0.311	−0.185	<0.001
Willingness to vaccinate child (aged 5–11 years)	0.012	−0.148	0.172	0.882
Willingness to vaccinate child (aged 12–18 years)	−0.305	−0.457	−0.152	<0.001
Number of children in the family	−0.043	−0.080	−0.007	0.021

*DV = mean complacency subscale score. Model R-squared = 0.452, adjusted R-squared = 0.447*.

#### Constraints

Table 3 shows the multivariate linear regression of constraints construct against vaccination. Male parents had on average less constraints to vaccination, as did parents who reported higher commitment to precautionary measures against COVID-19. Higher confidence and collective responsibility correlated with lower constraints, but higher complacency and calculation correlating with a higher constraints score. However, unlike confidence or complacency, willingness to vaccinate children of any age group was not significantly associated with a change in perceived constraints against vaccination.

**Table 3:** Multivariate linear regression analysis of parents' constraints as scored by the 5C scale.

	**Unstandardized beta coefficients**	**95.0% CI for beta**		***p*-value**
		**Lower bound**	**Upper bound**	
(Constant)	3.833	3.352	4.315	<0.001
Gender = Male	−0.141	−0.260	−0.023	0.020
Age group	−0.025	−0.088	0.037	0.427
Parents educational level: university degree	0.370	−0.047	0.786	0.082
Parents' educational level: higher postgraduate studies	0.219	0.042	0.396	0.015
Households monthly income.	−0.042	−0.101	0.018	0.169
Employment= Yes	0.200	0.060	0.340	0.005
Parent did not receive the COVID-19 vaccine.	−0.243	−0.557	0.071	0.129
*Mean confidence score*	−0.089	−0.141	−0.037	0.001
*Mean complacency score*	0.268	0.223	0.314	<0.001
*Mean calculation score*	0.065	0.023	0.108	0.003
*Mean collective Responsibility score*	−0.325	−0.381	−0.269	<0.001
Willingness to vaccinate child (aged 5–11 years)	−0.107	−0.248	0.035	0.140
Willingness to vaccinate child (aged 12–18 years)	−0.016	−0.152	0.120	0.814
Perceived overall commitment to universal precautionary measures against COVID-19	−0.058	−0.113	−0.002	0.042
Use of CDC website as a source of information	−0.147	−0.287	−0.007	0.039
Use of medical articles as a source of information	−0.149	−0.266	−0.032	0.012

*DV = mean complacency subscale score. Model R-squared = 0.484, adjusted R-squared = 0.480*.

#### Calculation

Table 4 shows the multivariate linear regression of parents' reported calculation. The construct of calculation measured the parents' engagement in vaccine-related information searching. Certain factors were associated with a significant increase in engaging in extensive information searching regarding vaccines. These factors included higher complacency, constraints, and collective responsibility scores, having more children in the household, and, predictably, reporting the use of specialized sources of information on COVID-19 such as medical articles or CDC websites. The only two variables which were associated with low calculation scores were being a Saudi national and reporting willingness to vaccinate children aged 5–11 years.

**Table 4:** Multivariate linear regression analysis of parents' calculation as scored by the 5C scale.

	**Unstandardized beta coefficients**	**95.0% CI for B**		***p*-value**
		**Lower bound**	**Upper bound**	
(Constant)	3.383	2.695	4.071	<0.001
Gender = Male	−0.143	−0.288	0.002	0.053
Age group	0.014	−0.065	0.092	0.736
Nationality: Saudi	−0.246	−0.417	−0.076	0.005
Households' monthly income	0.050	−0.021	0.122	0.168
Family member previously infected with COVID-19	−0.063	−0.181	0.054	0.289
*Mean confidence score*	−0.029	−0.095	0.036	0.376
*Mean complacency score*	0.197	0.138	0.256	<0.001
*Mean constraints score*	0.092	0.025	0.159	0.007
*Mean collective responsibility score*	0.129	0.057	0.200	<0.001
Willingness to vaccinate child (aged 5–11 years)	−0.211	−0.388	−0.035	0.019
Willingness to vaccinate child (aged 12–18 years)	0.000	−0.171	0.170	0.997
Number of children in the family	0.061	0.020	0.102	0.004
Perceived overall commitment to universal precautionary measures against COVID-19	0.061	−0.010	0.132	0.090
Use of MOH website as a source of information	0.170	−0.009	0.348	0.062
Use of CDC website as a source of information	0.258	0.083	0.433	0.004
Use of Videos such as YouTube as source of information	0.222	−0.001	0.445	0.051
Use of medical articles as a source of information	0.207	0.060	0.355	0.006

*DV = mean complacency subscale score. Model R-squared = 0.114, adjusted R-squared = 0.102*.

### Collective responsibility

Table 5 details the multivariate linear regression analysis of the construct of collective responsibility. Parents with postgraduate high degree and those willing to vaccinate their 12–18 years child correlated with higher collective responsibility scores. Other psychological antecedents of vaccination, confidence and calculation also correlated significantly with higher collective responsibility scores, while complacency and constraints correlated with a significant decrease in collective responsibility scores.

**Table 5:** Multivariate linear regression analysis of parents' collective responsibility as scored by the 5C scale.

	**Unstandardized beta coefficients**	**95.0% CI for beta**		***p*-value**
		**Lower bound**	**Upper bound**	
(Constant)	**4.187**	3.770	4.603	<0.001
Gender = Male	−0.064	−0.172	0.043	0.241
Age group	−0.017	−0.077	0.042	0.565
Parents' educational level: higher postgraduate studies	0.235	0.076	0.395	0.004
Employment status = employed	0.077	−0.049	0.203	0.231
Parent previously infected with COVID-19	0.081	−0.032	0.194	0.160
Family member previously infected with COVID-19	−0.068	−0.165	0.029	0.168
Parent did not receive the COVID-19 vaccine.	−0.949	−1.236	−0.663	<0.001
*Mean confidence score*	0.422	0.379	0.465	<0.001
*Mean complacency score*	−0.172	−0.215	−0.129	<0.001
*Mean constraints score*	−0.278	−0.325	−0.231	<0.001
*Mean calculation score*	0.080	0.040	0.119	<0.001
Has children aged 12–18 years	−0.128	−0.231	−0.025	0.015
Willingness to vaccinate child (aged 5–11 years)	−0.007	−0.138	0.124	0.920
Willingness to vaccinate child (aged 12–18 years)	0.196	0.068	0.324	0.003
Use of MOH website as a source of information	0.084	−0.048	0.216	0.210
Use of medical articles as a source of information	−0.102	−0.207	0.002	0.054

*DV = mean complacency subscale score. Model R-squared = 0.640, adjusted R-squared = 0.636*.

## Discussion

In this study, we investigated parents' COVID-19-related history, willingness to vaccinate their children, and psychological antecedents of vaccination amidst the rapid spread of the Omicron variant. We found high scores of collective responsibilities amongst our respondents, and lower complacency and constraints scores. The relationship between the five psychological antecedents of vaccination in our sample was consistent with how they are predicted to influence vaccination behavior. The parental psychological antecedents of vaccination were significantly associated with multiple sociodemographic and COVID-19-related factors, including their used sources of information on COVID-19. Additionally, we found that a large proportion of parents were unwilling to vaccinate their children against COVID-19. Those who were willing to vaccinate their children had on average higher confidence in vaccines, lower complacency about COVID-19, and higher sense of collective responsibility.

To the best of our knowledge, this is the first study in KSA to explore the general public's perceptions of COVID-19 and its vaccination amidst the Omicron variant surge. Moreover, it is the first study to utilize the 5C psychological antecedents of vaccination scale to determine the parents' vaccine hesitancy during the Omicron variant emergence. Our participants are comparable to previously published studies from the region (36, 62, 63, 65, 66), and the predominant age group follows the population trend of KSA according to the latest population estimates (67). Most respondents in our study reported no willingness to vaccinate any of their children with the COVID-19 vaccine, with only one-third of parents reporting willingness to vaccinate younger children aged 5–11 years. A study investigating parental willingness to vaccinate their children in KSA almost a year prior to this report revealed similarly that less than half of the respondents were willing to vaccinate their children (68).

The 5C Scale was previously used in the Arab population including KSA to explore vaccine acceptance and intentions during the COVID-19 pandemic (56). Results in the present study revealed that the highest mean score for any of the antecedents of vaccination was for collective responsibility. This suggests that our respondents had a sense of moral, ethical, and societal responsibility to protect others by vaccinating themselves and contributing to herd immunity. Collective responsibility has been associated with increased vaccine acceptance in previous reports (69). For example, previous research has shown that increased vaccine acceptance in Japan was associated with the need for personal and public protection as well as having a sense of collective responsibility (70). Another study showed that of the 5Cs, the highest predictors for COVID-19 vaccine acceptance were collective responsibility and confidence (55).

On the other hand, the second highest score in our sample was for the construct of calculation, which has a negative impact on vaccine acceptance. An earlier study showed that constraints and calculation were associated with lower vaccine intake (55). Another study also showed that calculation and constraints were negatively associated with the intent to vaccinate against COVID-19. Our sample's lowest score, on average, was for the construct of constraints followed by complacency about COVID-19. This ranking of constructs suggests a relatively lower complacency about COVID-19, and higher sense of responsibility and confidence in vaccines. Overall, it is important to keep track of the public's confidence and complacency about COVID-19, especially that a large North American study demonstrated that these two factors explained between 38 and 21% of the variation in COVID-19 vaccine hesitancy (71).

In the previous study conducted in the Arab world, the researchers used cut-off scores to divide respondents into two groups, labeled Yes and No, for each of the psychological antecedents of the 5C (56). In their analysis, the highest reported antecedent by category in KSA was confidence, followed by collective responsibility, calculation, complacency, and finally constraints. Their results suggested that respondents in KSA showed high confidence in COVID-19 vaccines, when compared to other countries, as was found in UAE and Kuwait (56). That pattern likely represented the active vaccination programs in these countries. Our current study did not utilize the same cut-off scores. Nevertheless, using the mean score of each construct in the 5C scale, confidence ranked third in our sample after collective responsibility and calculation. This change in reported confidence from that previous study may indicate that confidence in vaccines in KSA has been affected by the emergence of new variants, and possibly reflects the public's response after the introduction of vaccines to younger age groups.

The revealed correlations between the different psychological antecedents of vaccination in parents in our sample are similar to the originally proposed constructs and how they should influence one another (51). The positive relationship between confidence and collective responsibility, and their negative correlation with complacency and constraints, is consistent with the theoretical underpinnings of the psychological antecedents of vaccination. The weaker correlation between calculation and the other constructs possibly illustrated how calculation represents a pattern of risk-assessment that only informs vaccination behavior depending on the sources of information used. The theoretical justification of calculation as an antecedent of vaccination was discussed at length in the original paper that introduced the 5C scale (51).

In addition, our correlation analysis between the reported commitment to precautionary measures against COVID-19 and the 5C constructs demonstrated how collective responsibility, for example, is correlated with precautionary behavior against COVID-19 and not only with vaccination behavior. Since most individuals in KSA reported practicing good precautionary measures against SARS-CoV-2 back in August 2020 (72), our findings shed light on the public's commitment to preventative measures against COVID-19 by relating it to vaccination behavior as the pandemic evolved in our region. In fact, the strong relationship between poor compliance to governmental precautions against COVID-19 and negative views about vaccines has been shown in a large panel study of adults in the UK during the COVID-19 pandemic.

Further regression analysis of the 5C subscale scores showed that the psychological antecedents of vaccination are significantly correlated with sociodemographic and COVID-19-related factors. It is important to note that these correlations were found in the context of a rising COVID-19 case count and widespread Omicron infections. We found that reporting willingness to vaccinate children of the two specified age groups (5–11 and 12–18 years) was significantly correlated with the 5C constructs. Confidence in vaccines was predictably associated with willingness to vaccinate children in both age groups. This supports the concept of confidence and its relationship with vaccine intention (51). It is also in agreement with previous reports using the 5C scale during the COVID-19 pandemic in different countries (69, 73). Complacency about COVID-19 in our sample was correlated with decreased willingness to vaccinate children, but only those aged 12–18 years. Indeed, complacency indicates that parents view vaccines as unnecessary, and that the disease is not serious or risky (51). This may explain why, in our sample, parents who reported not taking the annual flu vaccine scored higher on complacency.

Furthermore, we found that perceived constraints by the responding parents were not correlated with willingness to vaccinate their children. The construct of constraints is theorized to capture psychological and structural barriers to vaccination (such as time availability or geographical accessibility) (51). Therefore, it is supposed to correlate with reduced vaccination behavior, but not necessarily intention to vaccinate. Indeed, constraints against vaccination may represent barriers against translating vaccination intentions into behavior (73). Moreover, calculation was correlated with reduced willingness to vaccinate children in the 5–11 age group. Excessive information gathering may predict decreased intention to vaccinate because people who calculate more are more likely to be risk averse (51). But, as discussed previously, this depends on the quality of the information gathered and its sources. As for collective responsibility, it was correlated with increased willingness to vaccinate children aged 12–18 years, but no association with willingness to vaccinate younger children. In general, collective responsibility indicates willingness to protect others by vaccination self to achieve herd immunity (51), and has been shown to correlate with COVID-19 vaccination acceptance and intent to vaccinate (55, 69). Our results show that this sense of responsibility may extend to willingness to vaccinate one's own children against COVID-19, at least in the specified age group.

On a different note, parents' gender was not significantly correlated with any of the 5C constructs in our sample, except for higher perceived constraints in men. This could be due to national trends in work status but may represent a genuine difference in our sample as it persisted even when holding employment constant. However, gender has been found to be associated with vaccination behavior during the COVID-19 pandemic. For example, a study from the US demonstrated that female participants were more hesitant to vaccinate (74). In addition, the study exploring the psychological antecedents of individuals in 13 Arab countries prior to the emergence of Omicron, revealed that gender differences were present in confidence and calculation, with female respondents scoring lower on confidence but higher on calculation (56). This disparity between those findings and the present ones could be attributed to differences between the samples or due to the changing perceptions of people with the evolving pandemic.

In terms of age, we found older age groups were more likely to score higher on the confidence subscale of the 5C Scale. This was not found amongst healthcare workers in a culturally similar sample in Kuwait (55). This could be because the association between older age groups and confidence in vaccines may be relevant to the public and not to healthcare providers. Employment, including being a healthcare worker, was not significantly associated with any of the 5C construct besides constraints. This association suggested that those who are employed perceive, on average, more barriers to being vaccinated against COVID-19, which is consistent with the construct of constraints as it was originally introduced (73). Remarkably, the relationship we found between having postgraduate education and the psychological antecedents of COVID-19 vaccination has been reported in the literature (56).

## Study limitations

While this study explored the parental concepts during the first month of the Omicron variant surge, it has few limitations related to the cross-sectional design, convenient sampling, and possible recall bias. The convenient sampling could have selection bias, over-or under-representation of the population and non-generalizability potentials. Also, the actual response rate could not be calculated due to the various social media platforms that were used to distribute our survey. The current study showed that 54% were unwilling to vaccinate their children aged 5–11, and 57.2% were unwilling to give the additional booster vaccine to children aged 12–18. This finding may be related to the fact that the current generation of vaccines is less protective against Omicron and its subvariants. In one study, a two-dose vaccine of mRNA showed an effectiveness of 65.5% at 2–4 weeks after the second dose, 15.4% after 15–19 weeks and was only 8.8% after that (75). Thus, the interpretation of the overall willingness of the parents to vaccinate children may have been affected by such data and need to be interpreted as such. While the study is among the first to explore the public's perceptions toward the novel SARS-CoV-2 Omicron variant, perceptions are likely to change as more data about this variant emerges over time. More research could explore this evolving situation to enrich the literature on emerging SARS-CoV-2 variants and future infectious disease outbreaks.

## Conclusions

During the early period of Omicron SARS-CoV-2 variant spread, we found high scores of collective responsibilities amongst parents, and lower complacency and constraints scores. Parents willing to vaccinate their children had higher confidence in vaccines, lower complacency about COVID-19, and a higher sense of collective responsibility. The large proportion of parents who were unwilling to vaccinate their children against COVID-19 could signal vaccination avoidance in the face of increasing COVID-19 variants spread. Addressing vaccine worry and the impact of COVID-19 on individuals and the public must be investigated further.

## Data availability statement

The original contributions presented in the study are included in the article/Supplementary material, further inquiries can be directed to the corresponding author.

## Ethics statement

The study was approved by the Institutional Review Board of King Saud University, Riyadh, Saudi Arabia (approval 21/01039/IRB). The patients/participants provided their written informed consent to participate in this study.

## COVID-19 Saudi research consortium

Abdulkarim Alrabiaah, Ali Alhaboob, Amr Jamal, Yazed Ahmed Alkriadees, Ali A. Rabaan, Fahad AlZamil.

## Author contributions

SAle, MA, M-HT, AA-E, IE, BS, FAlj, KA, SAls, MB, ZM, and JA-T conceptualized the study, analyzed the data, and wrote the manuscript. RA, RB, FAls, NA, RH, KS, and SM contributed to the study design, collected, analyzed, interpreted data, and edited the manuscript. All authors reviewed and approved the final version of the manuscript.

## Funding

This project was funded by Princess Nourah Bint Abdul Rahman University researchers supporting project (number PNURSP2022R21), Princess Nourah Bint Abdul Rahman University, Riyadh, Saudi Arabia.

## Conflict of interest

The authors declare that the research was conducted in the absence of any commercial or financial relationships that could be construed as a potential conflict of interest.

## Publisher's note

All claims expressed in this article are solely those of the authors and do not necessarily represent those of their affiliated organizations, or those of the publisher, the editors and the reviewers. Any product that may be evaluated in this article, or claim that may be made by its manufacturer, is not guaranteed or endorsed by the publisher.

## Abbreviations

5C Scale: The 5C psychological antecedents of vaccination scale
CDC: Centers for disease control and prevention
COVID-19: Coronavirus disease 2019
HCW: Healthcare workers
MoH: Ministry of health
SARS-CoV-2: Severe acute respiratory syndrome coronavirus 2
WHO: World health organization.
