# Supplementary material for: Parental perceptions and the 5C psychological antecedents of COVID-19 vaccination during the first month of omicron variant surge: A large-scale cross-sectional survey in Saudi Arabia
Source: Front Pediatr. 2022 Aug 16;10:944165. doi: 10.3389/fped.2022.944165 (PMC9424678; doi:10.3389/fped.2022.944165)
Supplement: Supplementary file 1 [file Table_1.docx]

| **Table. A1: Descriptive analysis of the parents’ sociodemographic characteristics.** | | |
| --- | --- | --- |
|  | Frequency | Percentage |
| **Gender** |  |  |
| Female/mother | 875 | 65.3 |
| Male/father | 465 | 34.7 |
| **Age group** |  |  |
| 25-34 years | 267 | 19.9 |
| 35-44 years | 628 | 46.9 |
| 45-54 years | 308 | 23.0 |
| 55-64 years or older | 137 | 10.2 |
| **Nationality** |  |  |
| Saudi | 1067 | 79.6 |
| Non-Saudi | 273 | 20.4 |
| **Educational Level** |  |  |
| High school or less | 165 | 12.3 |
| University Degree | 1024 | 76.4 |
| Higher postgraduate studies | 151 | 11.3 |
| **Household monthly income** |  |  |
| Prefer not to answer/unemployed | 59 | 4.4 |
| Less than 5000 SR | 243 | 18.1 |
| 5000-10000 SR | 204 | 15.2 |
| More than 10000 SR | 834 | 62.2 |
| **Employment** |  |  |
| Unemployed/Retired | 292 | 21.8 |
| Freelance | 110 | 8.2 |
| Healthcare worker | 308 | 23.0 |
| Employee | 630 | 47.0 |
| **Residence** |  |  |
| Central region | 885 | 66 |
| Northern region | 82 | 6.1 |
| Eastern region | 123 | 9.2 |
| Southern region | 48 | 3.6 |
| Western region | 202 | 15.1 |
