# Supplementary material for: Parental perceptions and the 5C psychological antecedents of COVID-19 vaccination during the first month of omicron variant surge: A large-scale cross-sectional survey in Saudi Arabia
Source: Front Pediatr. 2022 Aug 16;10:944165. doi: 10.3389/fped.2022.944165 (PMC9424678; doi:10.3389/fped.2022.944165)
Supplement: Supplementary file 2 [file Table_2.docx]

| **Table A2: Parents’ Psychological Antecedents of Vaccination as measured by the 5C Scale items.** | | | |
| --- | --- | --- | --- |
|  | **M** | **SD** | ***Rank*** |
| **CONFIDENCE** |  |  |  |
| 1. I am completely confident that vaccines are safe. | 4.28 | 1.85 | ***3*** |
| 2. Vaccinations are effective. | 4.59 | 1.71 | ***2*** |
| 3. Regarding vaccines, I am confident that public authorities decide in the best interest of the community. | 5.23 | 1.67 | ***1*** |
| **COMPLACENCY** |  |  |  |
| 4. Vaccination is unnecessary because vaccine-preventable diseases are not common anymore. | 3.09 | 1.74 | ***3*** |
| 5.My immune system is so strong; it also protects me against diseases | 3.89 | 1.76 | ***1*** |
| 6.Vaccine-preventable diseases are not so severe that I should be vaccinated | 3.43 | 1.85 | ***2*** |
| **CONSTRATINTS** |  |  |  |
| 7. Everyday stress prevents me from being vaccinated. | 2.50 | 1.55 | ***2*** |
| 8. For me, it is inconvenient to be vaccinated. | 2.99 | 1.94 | ***1*** |
| 9. Visiting the doctor makes me feel uncomfortable; this keeps me from being vaccinated. | 2.31 | 1.51 | ***3*** |
| **CALCULATION** |  |  |  |
| 10. When I think about being vaccinated, I weigh its benefits and risks to make the best decision possible. | 4.90 | 1.78 | ***3*** |
| 11. For each and every vaccination, I closely consider whether it is useful for me. | 5.37 | 1.46 | ***2*** |
| 12. It is important for me to fully understand the topic of vaccination before I get vaccinated. | 5.63 | 1.44 | ***1*** |
| **COLLECTIVE RESPONSIBILITY** |  |  |  |
| **13. When everyone else is vaccinated, I don’t have to be vaccinated, too. | 3.01 | 1.84 | ***3*** |
| 14. I get vaccinated because I can also protect people with a weaker immune system. | 5.37 | 1.66 | ***2*** |
| 15. Vaccination is a collective action to prevent the spread of disease. | 5.62 | 1.58 | ***1*** |
| *M=mean, SD= standard deviation. Rank= descending mean rank. *Item13 was reverse coded to align its magnitude with the other collective responsibility indicators when the composite score was computed because it is a negatively worded statement.* | | | |
