# Supplementary material for: Parental perceptions and the 5C psychological antecedents of COVID-19 vaccination during the first month of omicron variant surge: A large-scale cross-sectional survey in Saudi Arabia
Source: Front Pediatr. 2022 Aug 16;10:944165. doi: 10.3389/fped.2022.944165 (PMC9424678; doi:10.3389/fped.2022.944165)
Supplement: Supplementary file 3 [file Table_3.docx]

| **Table A3: Reliability analysis of the 5C's Questionnaire.** | | |
| --- | --- | --- |
|  | **Number of items** | **Cronbach's alpha** |
| Confidence (CNF) | 3 | 0.87 |
| Complacency (CPC) | 3 | 0.75 |
| Constraints (CNST) | 3 | 0.71 |
| Calculation (CLC) | 3 | 0.73 |
| Collective Responsibility (CR) | 3 | 0.83 |
