# Supplementary material for: Parental perceptions and the 5C psychological antecedents of COVID-19 vaccination during the first month of omicron variant surge: A large-scale cross-sectional survey in Saudi Arabia
Source: Front Pediatr. 2022 Aug 16;10:944165. doi: 10.3389/fped.2022.944165 (PMC9424678; doi:10.3389/fped.2022.944165)
Supplement: Supplementary file 4 [file Table_4.docx]

| **Table A4: Bivariate correlations between the parents’ 5C Psychological Antecedents of Vaccination** | | | | | |
| --- | --- | --- | --- | --- | --- |
|  | **CNF** | **CPC** | **CNST** | **CLC** | **CR** |
| **Confidence (CNF)** | 1 |  |  |  |  |
| **Complacency (CPC)** | -.506^**^ |  |  |  |  |
| **Constraints (CNST)** | -.536^**^ | .580^**^ |  |  |  |
| **Calculation (CLC)** | -.133^**^ | .229^**^ | .165^**^ |  |  |
| **Collective Responsibility (CR)** | .723^**^ | -.568^**^ | -.615^**^ | -.087^**^ |  |
| **Perceived Family overall Commitment to Universal precautions.** | .089^**^ | -.096^**^ | -.120^**^ | .066^*^ | .116^**^ |
| *****. p-value <= 0.01 level (2-tailed). *. p-value<= 0.05 level (2-tailed).*** | | | | | |
