# Supplementary material for: Parental perceptions and the 5C psychological antecedents of COVID-19 vaccination during the first month of omicron variant surge: A large-scale cross-sectional survey in Saudi Arabia
Source: Front Pediatr. 2022 Aug 16;10:944165. doi: 10.3389/fped.2022.944165 (PMC9424678; doi:10.3389/fped.2022.944165)
Supplement: Supplementary file 5 [file Data_Sheet_1.PDF]

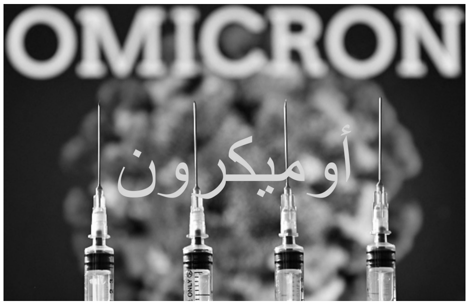

متحور اوميكرون و تقييم قبول البالغين و الوالدين لتطعيم اطفالهم ضد فيروس الكوفيد

Omicron and Children COVID19 Vaccine Perceptions among Parents

أنت مدعو للمشاركة في هذه الدراسة عن رأي البالغين او الوالدين بالنسبة لمتحور اوميكرون وتطعيم الأطفال للكوفيد سنوياً

الدراسة موافق عليها من لجنة الأبحاث في جامعة الملك سعود و مشاركتك فيها طوعية حيث نهدف من خلالها لمعرفة رأي البالغين او الوالدين بالنسبة لتطعيم الكوفيد لهم او أطفالهم(5-18) سنة ضد الكوفيد

متابعتك في الاستبيان تعني موافقتك على المشاركة في ه>ه الدراسةز

و لمزيد من المعلومات يمكنك التواصل مع الباحثين الرئيسين
COVID-19 Research Consortium
mtemsah@ksu.edu.sa

You are invited to participate in this study which assesses the factors affecting parents' acceptance and perceptions of annual COVID vaccines.

The study was approved by the research comity of King Saud University and your participation is voluntary, we aim of this study is to assess the factors affecting parents' acceptance and perceptions of the COVID-19 vaccine in children.

The study was approved by the research committee of King Saud University and your participation is voluntary, we aim from this study to research the social and psychological factors affecting children during the COVID-19 pandemic.

Completing the survey means you voluntarily accept to participate in the study.

For more information, you can email the principal investigator: the COVID-19 Research Consortium

mtemsah@ksu.edu.sa

* 1. ألشخص المجيب:

Respondant:

- ◯ Mother الأم
- ◯ Father الأب
- ◯ فرد اخر من العائلة : يرجى تحديد الصلة
  Other relative: please specify

* 2. هل أخذت تطعيم الكوفيد أنت؟

Did you take the COVID vaccine yourself?

- ◯ Yes: with the booster third dose نعم: مع الجرعة المنشطة الثالثة
- ◯ No due to a medical exception لا : بسبب استثناء طبي
- ◯ No: I do not believe in the Covid vaccine لا: انا غير مقتنع بلقاح الكوفيد
- ◯ (لا) بسبب آخر
  No : other cause (please specify why)

* 3. مدى التزام الأسرة مع توصيات الاحترازية لكوفيد (مثل ارتداء الكمامة و التباعد الاجتماعي و تجنب الأماكن المزدحمة)

How is the family's commitment to COVID precautions recommendations (like wearing masks and social distancing and avoiding crowds)

Rarely committed ملتزم نادراً	Somewhat committed ملتزم احيانا	Medium commitment التزام متوسط	Usually committed عادة ملتزم	Always committed ملتزم دائماً
★	★	★	★	★

* 4. في اي منطقة تسكن؟

Region where you live?

- ◯ Central region المنطقة الوسطى
- ◯ Northern region المنطقة الشمالية
- ◯ Eastern region المنطقة الشرقية
- ◯ Another country بلد آخر
- ◯ Southern region المنطقة الجنوبية
- ◯ Western region المنطقة الغربية

* 5. Ageالعمر

- ◯ 18-24
- ◯ 25-34
- ◯ 35-44
- ◯ 45-54
- ◯ 55-64
- ◯ 65+

* 6. Parent’s education: المستوى التعليمي :

- ◯ Primary school ابتدائي
- ◯ Middle school متوسط
- ◯ Highschool ثانوي
- ◯ University جامعي
- ◯ Others: please specify
  غير ذلك : يرجى التحديد

[ ]

* 7. متوسط الدخل الشهري للعائلة:
Mean family’s monthly income

- ◯ Less than 5000 SR أقل من
- ◯ 10000-5000 ريال SR
- ◯ 10001-15000 ريال SR
- ◯ More than 15000 SR أكثر من
- ◯ Prefer not to answer افضل عدم الإجابة

* 8. Nationality الجنسية:

- ◯ Saudi سعودي
- ◯ Non Saudi غير سعودي

* 9. Job-status العمل؟

- ◯ Freelance اعمال حرة
- ◯ Healthcare worker قطاع صحي
- ◯ employee موظف - موظفة
- ◯ Others: please specify
  غير ذلك: يرجى التحديد
- ◯ teacher مدرس - مدرسة
- ◯ Unemployed لا اعمل حاليا

[ ]

* 10. هل اصبت انت بكوفيد سابقا؟
Were you affected by COVID-19 yourself?

- ◯ No لا
- ◯ Yes, but did not require hospitalization نعم لكن لم احتاج التنويم بالمستشفى
- ◯ Yes, and required hospitalization نعم و احتجت التنويم بالمستشفى
- ◯ Yes, and required hospitalization and ICU نعم و احتجت التنويم بالمستشفى في العناية المركزة

* 11. هل تحصل على تطعيم (لقاح) الإنفلوزا سنويا
Do you take the flu vaccine every year?

- ◯ Yes نعم
- ◯ No لا

12. هل تؤيد الحصول على جرعة منشطة ثانية من تطعيم الكوفيد في 2022؟
Do you support receiving a second booster Covid vaccine in 2022?

- ◯ Yes نعم
- ◯ No لا

* 13. مقارنة بمتحور الكوفيد السابق (دلتا في عام 2021)، كم تقدرمستوى القلق لديك حالياً من متحور الأوميكرون (2022)؟
Compared to the previous Delta COVID mutation in 2021, how much are you worried nowadays in 2022 about the Omicron variant nowadays?

- ◯ Much worried with the Omicron قلق اكثر بسبب اوميكرون
- ◯ Same worry level نفس مستوى القلق
- ◯ Less worried with the Omicron قلق اقل بسبب اوميكرون

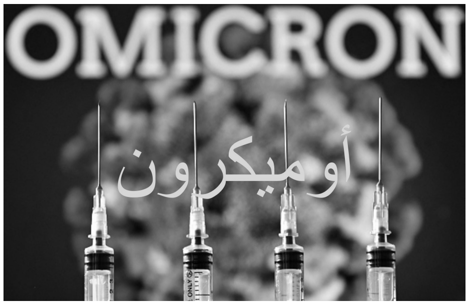

متحور اوميكرون و تقييم قبول البالغين و الوالدين لتطعيم اطفالهم ضد فيروس الكوفيد

* 14. لماذا تؤيد الحصول على جرعة منشطة ثانية من تطعيم الكوفيد في 2022؟
(الرجاء اختيار كل ما ينطبق)

Why do you support receiving a second booster Covid vaccine in 2022?
(Please choose all that apply)

- ☐ Worried Omicron might surge to cause national lockdown الزيادة المتسارعة في حالات الأوميكرون قد تؤدي الى اعادة الحجر
- ☐ The previous Covid vaccine decreses over few months فعالية التطعيمات السابقة تنقص بعد بضعة شهور
- ☐ another worldwide pandemic موجة ثانية من الجائحة
- ☐ me or my family being affected by the COVID Omicron disease ان اصاب أنا او احد افراد اسرتي بمرض الكوفيد اوميكرون
- ☐ Other (please specify) اسباب أخرى (يرجى تحديدها)

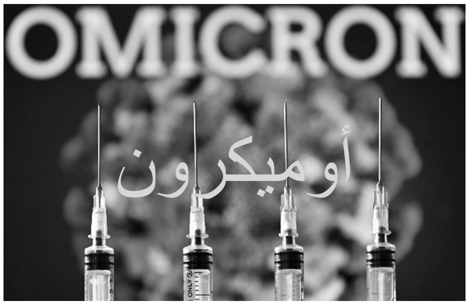

متحور اوميكرون و تقييم قبول البالغين و الوالدين لتطعيم اطفالهم ضد فيروس الكوفيد

* 15. لماذا لا تؤيد الحصول على جرعة منشطة ثانية من تطعيم الكوفيد في 2022؟
(الرجاء اختيار كل ما ينطبق)

Why you do NOT support receiving a second booster Covid vaccine in 2022?
(Please choose all that apply)

- ☐ Omicron causes mild disease الأوميكرون اعراضه خفيفة
- ☐ The previous Covid vaccine effects last for a year or more فعالية التطعيمات السابقة تستمر لمدة سنة أو اكثر
- ☐ the pandemic is over الجائحة انتهت
- ☐ I was affected by the COVID disease and so I have natural immunity لقد اصبت أنا بمرض الكوفيد سابقا و عندي مناعة له
- ☐ Other (please specify) اسباب أخرى (يرجى تحديدها)

[ ]

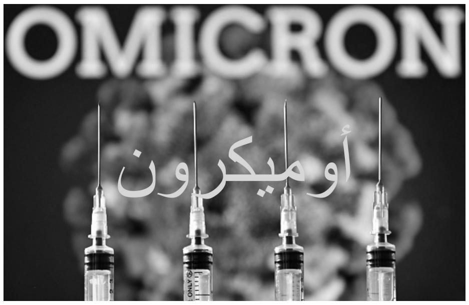

متحور اوميكرون و تقييم قبول البالغين و الوالدين لتطعيم اطفالهم ضد فيروس الكوفيد

* 16. Number of your children عدد أطفالك

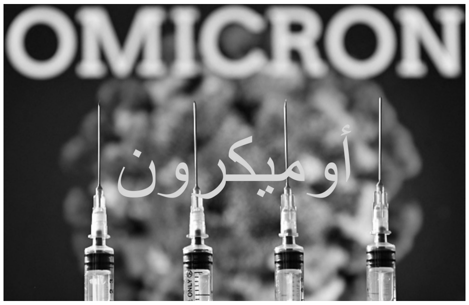

متحور اوميكرون و تقييم قبول البالغين و الوالدين لتطعيم اطفالهم ضد فيروس الكوفيد

* 17. Do you have a child whose age is between 5-11 years
هل لديك طفل عمره بين 5-11 سنة؟

- ◯ Yes نعم
- ◯ No لا

* 18. هل لديك طفل عمره بين 12-18 سنة؟

Do you have a child whose age is between 12-18 years?

- ◯ Yes نعم
- ◯ No لا

* 19. هل تم تشخيص اي من اطفالك باي امراض عضوية او نفسية؟

Was any of your children ever diagnosed with an organic or psychological illness?

- ◯ No لا
- ◯ نعم: يرجى ذكر اسم المرض
  Yes: please specify disease

* 20. هل أنت على استعداد لتطعيم طفلك الذي يبلغ من العمر 5-11 عامًا ضد الكوفيد؟

Are you willing to vaccinate your 5-11 years old child for COVID-19?

- ◯ Yes نعم
- ◯ No: because vaccines aren’t safe لا: لأن اللقاحات ليست آمنة
- ◯ No: because I don’t think he’s at risk لا: لأنني لا أعتقد أنه في خطر من كوفيد
- ◯ No: not applicable ليس لدي طفل بهذا العمر

* 21. هل تنوي اعطاء جرعة تنشيطية تطعيم الكوفيد لطفلك من 12-18 سنة؟

Are you willing to give the COVID Booster vaccine to your child(children) 12-18 years old?

- ◯ Yes نعم
- ◯ No لا

* 22. ما مدى معرفتك بالاجراءات الاحترازية في المدارس في حال تسجيل اصابة كوفيد بين الطلاب او المدرسين؟
How much are you aware of the precautionary measures if there is a COVID case at school?

- ◯ Extremely familiar معرفة كاملة
- ◯ Somewhat familiar بعض المعرفة
- ◯ Not at all familiar لا اعرفها

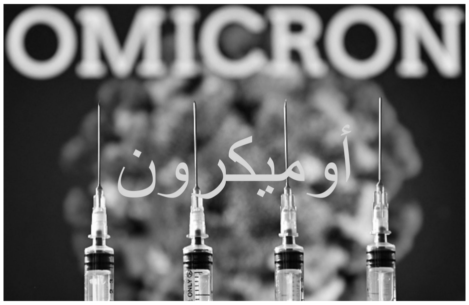

متحور اوميكرون و تقييم قبول البالغين و الوالدين لتطعيم اطفالهم ضد فيروس الكوفيد

اسباب رفض الجرعة التنشيطية من تطعيم كوفيد للأطفال

* 23. اختررت عدم اعطاء طفلك الجرعة التنشيطية من لقاح كوفيد
ما هي اسبابك؟
(اختر كل ما ينطبق)

You choose not to give the Booster COVID Vaccine to your child:
What are your reasons for avoiding the Covid vaccine in children?
(Choose what apply)

☐ معلومات غير كافية حول سلامة اللقاح الجديد
Inadequate data about the safety of a new vaccine

☐ (أنا ضد اللقاح بشكل عام (أو أتجنب الأدوية كلما أمكن ذلك
I am against vaccine in general (or I avoid medications whenever possible)

☐ إعطاء اللقاح مؤلم أو غير مريح
Vaccine administration is painful or inconvenient

☐ لقد اصيب الطفل بالفعل بعدوى الكوفيد
My child already had a COVID infection

☐ قلق من الآثار الضارة للقاح
A concern of adverse effects of the vaccine

☐ قلق من الإصابة بمرض الكوفيد من اللقاح نفسه
A concern of acquiring Covid19 from the vaccine

☐ قلق من أن اللقاح غير فعال ضد الكوفيد المتحور
A concern of vaccine being ineffective from Covid mutations

☐ رد فعل سلبي مسبق للقاح
Prior adverse reaction to the vaccine

☐ أرى طفلي ليس في خطر كبير للإصابة بعدوى الكوفيد
I perceive my child as not at high risk to acquire Covid19 infection

☐ أرى أن طفلي ليس في خطر كبير للإصابة بمضاعفات إذا أصيب بعدوى الكوفيد
I perceive my child as not at high risk to develop complications if he/she get infected with Covid19

☐ (غير ذلك (يرجى التحديد
Other (please specify)

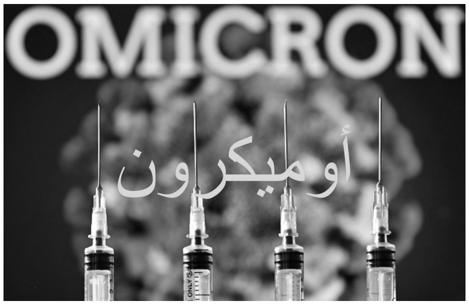

متحور اوميكرون و تقييم قبول البالغين و الوالدين لتطعيم اطفالهم ضد فيروس الكوفيد

* 24. ما هي مصادر معلوماتك عن الكوفيد بشكل عام؟
(اختر كل ما ينطبق عليك من فضلك)

What are your COVID information sources?
(Please choose all that apply)

- [ ] وزارة الصحة (الموقع الرسمي و وسائل التواصل الرسمية مثل تويتر وزارة الصحة)
Minstry of health (website, social media like twitter...)
- [ ] موقع منظمة الصحة العالمية
WHO website
- [ ] مراكز السيطرة على الأمراض والوقاية منها
CDC Website
- [ ] وسائل التواصل الاجتماعي الأخرى مثل تويتر و فيسبوك
Other social media like Twitter and Facebook
- [ ] فيديوهات مثل يوتيوب
Videos such as YouTube
- [ ] مقالات طبية
Medical articles
- [ ] غير ذلك (يرجى التحديد)
Other (please specify)

____

25. هل يجب أن يكون لقاح كوفيد السنوي في فصل الخريف مثل لقاح الإنفلونزا؟
Should the annual covid vaccine be in the autumn like the flu vaccine?

- ◯ Yes نعم
- ◯ No لا
- ◯ Not sure غير متأكد

26. هل يجب أن يشمل لقاح كوفيد القادم المتحورات الجديدة مثل الأوميكرون و الدلتا؟
Should the next COVID vaccine include the new variants (like Omicron and Delta)?

- ◯ Yes نعم
- ◯ No لا
- ◯ Not sure غير متأكد

27. من الذي يجب أن يحصل على لقاح الكوفيد كل سنة؟
Who should get a regular COVID vaccine every year?

- [ ] Healthcare workers (doctors, nurses,...) عمال الرعاية الصحية (الأطباء والممرضات ...)
- [ ] elderly كبار السن
- [ ] Oncology patients مرضى الأورام
- [ ] immune suppressed patients المرضى الذين يعانون من ضعف المناعة
- [ ] Diabetes patients مرضى السكر
- [ ] Hypertension patients مرضى ارتفاع ضغط الدم
- [ ] غير ذلك (يرجى التحديد)
Other (please specify)
- [ ] لا أحد ممن سبق ذكرهم
None of the above
- [ ] COPD patients مرضى الانسداد الرئوي المزمن
- [ ] Chronic Kidney Disease poatients مرضى الكلى المزمنة
- [ ] Asthma patients مرضى الربو
- [ ] people with obesity الأشخاص المصابون بالسمنة
- [ ] children الأطفال

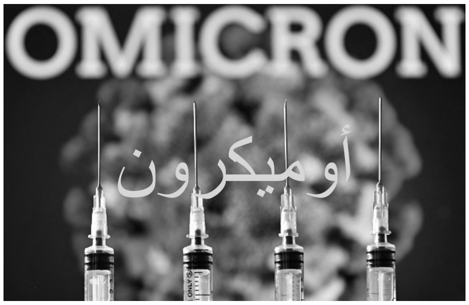

متحور اوميكرون و تقييم قبول البالغين و الوالدين لتطعيم اطفالهم ضد فيروس الكوفيد

* 28. من فضلك أجب على هذه الأسئلة الخاصة **بلقاحات فيروس كورونا و الأوميكرون**

Please answer these questions about the **Omicron and COVID-19 vaccine**:

	Strongly Disagree أرفض بشدة	Disagree أرفض	Somewhat Disagree أرفض إلى حد ما	Neutral محايد	Somewhat Agree أوافق إلى حد ما	Agree أوافق	Strongly Agree أوافق بشدة
أنا واثق تماما بأن اللقاحات آمنة I am completely confident that vaccines are safe.							
اللقاحات فعالة Vaccinations are effective.							
بالنسبة للقاحات ، أنا واثق من أن السلطات العامة تقرر ما هو أفضل لصالح المجتمع Regarding vaccines, I am confident that public authorities decide in the best interest of the community.							
اللقاح غير ضروري لأن الأمراض مثل الكوفيد التي يقي منها لم تعد منتشرة كما في السابق Vaccination is unnecessary because vaccine-preventable diseases are not common anymore.							
جهازي المناعي قوي جدا وأيضا يحميني من الأمراض My immune system is so strong; it also protects me against diseases							
الأمراض التي يمكن الوقاية منها باللقاح ليست خطيرة بالقدر الكافي لكي يتوجب عليّ الحصول على هذا اللقاح Vaccine-preventable diseases are not so severe that I should							

be vaccinated.							
يمنعني الضغط اليومي من الحصول على اللقاح<br>Everyday stress prevents me from being vaccinated.	○	○	○	○	○	○	○
بالنسبة لي ، من غير الملائم أخذ اللقاح<br>For me, it is inconvenient to be vaccinated.	○	○	○	○	○	○	○
أشعر بعدم الارتياح عند زيارة الطبيب، وهذا يجعلني أتجنب أخذ اللقاح<br>Visiting the doctor makes me feel uncomfortable; this keeps me from being vaccinated.	○	○	○	○	○	○	○
عندما أفكر بالحصول على اللقاح، أقارن ما بين المخاطر والفوائد لاتخاذ أحسن قرار متاح<br>When I think about being vaccinated, I weigh its benefits and risks to make the best decision possible.	○	○	○	○	○	○	○
بالنسبة لكل لقاح ، أفكر جيدا في فائدته بالنسبة لي<br>For each and every vaccination, I closely consider whether it is useful for me.	○	○	○	○	○	○	○
من المهم بالنسبة لي أن أفهم بشكل كامل كل ما يخص موضوع اللقاحات قبل أن أحصل على اللقاح<br>It is important for me to fully understand the topic of vaccination before I get vaccinated.	○	○	○	○	○	○	○
عندما يحصل الجميع على اللقاح، فأنا لست مضطرا لأخذ اللقاح أيضاً<br>When everyone else is vaccinated, I don’t have to be vaccinated, too.	○	○	○	○	○	○	○
أقوم بأخذ اللقاح لأوفر الحماية لأفراد المجتمع ذوي المناعة الأضعف<br>I get vaccinated because I can also protect people with a weaker immune system.	○	○	○	○	○	○	○

التطعيم هو عمل جماعي لمنع انتشار الأمراض Vaccination is a collective action to prevent the spread of disease.	○	○	○	○	○	○	○
